# A neutralizing monoclonal antibody (mAb A24) directed against the transferrin receptor induces apoptosis of tumor T lymphocytes from ATL patients

**DOI:** 10.1186/1742-4690-8-S1-A60

**Published:** 2011-06-06

**Authors:** Ivan C Moura, Yves Lepelletier, Bertrand Arnulf, Ali Bazarbachi, Renato C Monteiro, Olivier Hermine

**Affiliations:** 1 CNRS et Service d’hématologie, Hôpital Necker, Paris, France; 2 INSERM, hôpital Bichat, Paris, France; 3 Service d’hématologie, Hôpital Saint Louis, Paris, France; 4 Department of hematologiy, university of Beiruth, beiruth, Lebanon

## 

Adult T-cell leukemia/lymphoma (ATL) is an aggressive lymphoid proliferative disease that exists under diverse clinical forms ranging from chronic to acute. In contrast to resting T cells, human T-cell lymphotropic virus type 1 (HTLV-1) infected cells constitutively express high levels of surface transferrin receptor (TfR). Interestingly this expression is higher in acute than in chronic forms. We have characterized a new monoclonal antibody (mAb A24) directed against the human TfR that blocks the proliferation and induced apoptosis through mitochondria depolarization of ATL cells ex vivo. We determined that A24 binds TfR with an equilibrium constant (Kd) of 2.7 nM and competes with transferring for binding to TfR. Interestingly A24 exhibits an higher affinity than transferin when TfR are highly expressed. A24 inhibited [55Fe]-transferrin uptake through TfR endocytosis via the clathrin adaptor protein-2 complex pathway followed by transport to lysosomal compartments. In monkey administration of single and repeated doses of A24 did not induce significant toxicity except a slight decreased of haemoglobin level, increased of transferin and decreased of iron serum levels. Interestingly in lymph nodes, apoptosis was observed in germinal center in zone of high proliferation of B and T cells. Therefore, A24 might be a safe and effective treatment of ATLL particularly acute forms.

